# Parents’ social comparisons and adolescent self-esteem: the mediating effect of upward social comparison and the moderating influence of optimism

**DOI:** 10.3389/fpsyg.2025.1473318

**Published:** 2025-01-22

**Authors:** Hongyang Liu, Jana Kvintova, Lucie Vachova

**Affiliations:** Department of Psychology and Abnormal Psychology, Faculty of Education, Palacky University Olomouc, Olomouc, Czechia

**Keywords:** parents’ social comparison, upward social comparison, self-esteem, optimism, adolescent

## Abstract

Parents’ social comparison is a common phenomenon that occurs in China. It refers to the behavior of parents imagining other people’s children as an excellent role model without shortcomings and comparing their own children with them. This behavior may affect their child’s mood. The purpose of this study was to explore the impact of parents’ social comparison on adolescents’ self-esteem through the mediating role of upward social comparison, and to explore the moderating role of optimism in upward social comparison and self-esteem. Parents’ social comparison questionnaire, upward social comparison questionnaire, self-esteem questionnaire and life orientation questionnaire were distributed through online questionnaires, and 576 valid questionnaires were received. The results of the study found that upward social comparison plays a partial mediating role in the negative impact of parents’ social comparison on self-esteem, and optimism plays a moderating role in the impact of upward social comparison on self-esteem. This study illustrates the harmful effects of parents’ social comparison on adolescent mental health, but this harm can be mitigated through the teaching of optimism. This study shows that parents’ social comparison is not advisable, and attention should be paid to maintaining the optimistic attitude of teenagers to ensure their healthy growth.

## 1 Introduction

In recent years, the psychological well-being of Chinese adolescents has garnered increasing concern amidst rapid socio-economic changes and heightened educational pressures. Studies indicate a worrying trend of declining self-esteem among this demographic, a crucial aspect of adolescent development that influences future mental health outcomes. The social ecological system theory believes that the formation of self-esteem of adolescents is the result of their interaction with family, school, and society (Franco and Levitt, 1998). Family is the initial place of life for individuals. Parental parenting styles have an important impact on adolescents’ self-esteem. Wrong parenting styles can cause damage to a teenager’s self-esteem (Martinez et al., 2020; Szkody et al., 2021). Most previous studies have focused on the impact of family factors on adolescents’ self-esteem, for example, the parenting style (Aziz et al., 2021; Jannah et al., 2022; Peng et al., 2021), family environment (Krauss et al., 2020; Uçar et al., 2020), and family functionality (Chen et al., 2020).

Fewer studies have examined the impact of specific parental behavioral events on adolescent self-esteem. In many East Asian countries, especially Chinese families, parents often use “other people’s children” to motivate their children. Many Chinese parents will imagine that “other people’s children” are role models who are excellent in all aspects, while their own children have various shortcomings. This seems to be a common thing in Chinese families (Liu, 2018). However, is this kind of motivation from “other people’s children” applicable to teenagers? According to the “Youth Blue Book: China Minors’ Internet Use Report (2022),” “Look at other people’s children” was selected as one of the “five sentences that minors dislike most from their parents (Yong et al., 2022).” It can be seen that “other people’s children” do not have any motivating effect on teenagers but have a negative impact on them.

This behavior of parents trying to use “other people’s children” to motivate their children was first called “other people’s children” information, which is inspired by social comparison proposed by Festinger in 1954 (Liu, 2018). Social comparison is the motivation for individuals to compare themselves with others in order to judge the correctness of their own opinions (Festinger, 1957). Among them, upward society, which means that individuals compare a certain aspect, such as a certain ability, wealth level or appearance, with other individuals who are better than themselves (van de Ven, 2017), makes it easier for individuals to realize the gap between themselves and others, and thus experience negative and negative emotions, such as depression (Appel et al., 2015; Aubry et al., 2024; Liu et al., 2017; McCarthy and Morina, 2020; Wang et al., 2020), anxiety (Li, 2019; McCarthy and Morina, 2020; Zheng et al., 2020), etc. The researcher first compiled a 21-item “Other People’s Children” questionnaire and found that this can positively affect adolescents’ upward comparison tendency, and thus have a negative impact on adolescents’ self-esteem (Liu, 2018). To give this phenomenon a more academic concept, later researchers changed it to parents’ social comparison (Lee et al., 2020). Parents’ social comparison is more likely to produce a contrast effect, which may cause teenagers to feel more pain or frustration and form a negative self-evaluation, which in turn affects the mental health of teenagers (Cao, 2023; Chen, 2022; Liu, 2018). Limited past studies have proven that parents’ social comparison can have a negative impact on adolescents’ mental health, but few studies have explored the protective factors that mitigate this negative effect. In summary, the aim of this study is to investigate the complex interplay between parents’ social comparison, adolescent upward social comparison, and adolescent self-esteem, with a specific focus on the mediating role of upward social comparison and the moderating role of optimism.

In the domain of social psychology, the impact of parental behavior on the development of children’s social comparison tendencies has been a significant area of study. Research suggests that parents who frequently engage in social comparisons are likely to model and reinforce similar behaviors in their children (Festinger, 1954; Gibbons and Buunk, 1999). Particularly, when parents compare their children to others, they inadvertently teach their children to gauge their own worth and achievements against their peers. This modeling effect is crucial in the formation of upward social comparison tendencies among adolescents, where children learn to look at peers who are perceived to perform better to assess their own social and academic standing (Gibbons and Buunk, 1999). Thus, it is hypothesized that parents’ social comparison will be positively related to adolescents’ upward social comparison tendency, suggesting that parental influence plays a pivotal role in how children come to understand and engage with their social world.

In addition, an extensive body of research within developmental psychology suggests that the social comparison processes facilitated by parents can significantly impact the self-esteem of adolescents. According to social comparison theory, individuals determine their own social and personal worth based on how they stack up against others (Festinger, 1954). When parents frequently compare their children to others, particularly in unfavorable terms, it can lead to a decrease in self-esteem as children may perceive themselves as less competent, successful, or worthy compared to their peers (Myers and Crowther, 2009). This notion is supported by findings from studies indicating that children and adolescents whose parents engage in high levels of social comparison often report lower self-esteem, as these comparisons typically emphasize the superiority of peers (Gentzler et al., 2010). Therefore, it is hypothesized that there is a negative relationship between parents’ social comparison and adolescents’ self-esteem, highlighting the potential detrimental effects of comparative evaluations within the familial context.

Upward social comparison, wherein individuals compare themselves to those perceived as better off or superior in some respects, can have profound effects on one’s self-esteem. According to social comparison theory, engaging in upward comparison can often lead to feelings of inadequacy and decreased self-worth, particularly when individuals perceive the comparison of others as vastly superior to themselves (Festinger, 1954). This effect is especially pronounced in adolescents, a group for whom identity formation and self-esteem are particularly malleable and susceptible to external influences (Gibbons and Buunk, 1999). Empirical studies further validate this theory, showing that adolescents who frequently engage in upward social comparisons tend to report lower levels of self-esteem, as these comparisons highlight deficiencies rather than strengths (Bergagna and Tartaglia, 2018; Fagundes et al., 2020; Jiang and Ngien, 2020; Lewis, 2021; Schmuck et al., 2019). Thus, it is hypothesized that upward social comparison serves as a mediating variable in the relationship between parents’ social comparison and adolescents’ self-esteem, positing that the impact of parental comparisons on adolescent self-esteem is mediated by the degree to which adolescents engage in upward comparisons.

The interplay between social comparison and psychological well-being is significantly influenced by individual differences in dispositional traits such as optimism. Optimism, characterized by the general expectation that good things will happen, is proposed to moderate the effects of social comparison on self-esteem. According to Carver and Scheier, optimism can buffer the negative effects of stressful experiences and enhance coping strategies in challenging situations, which may include social comparisons that are unfavorable (Scheier et al., 2021). Specifically, in the context of parental social comparisons, where parents compare their children to others, such comparisons might typically undermine self-esteem through mechanisms of upward social comparison. However, for adolescents who are more optimistic, the detrimental impact of these comparisons on self-esteem may be less pronounced due to their more positive outlook and adaptive coping responses (Seligman, 2018; Sharma, 2022). Thus, it is hypothesized that optimism will moderate the relationship between upward social comparison (mediated by parents’ social comparison) and self-esteem, potentially reducing the negative impact of unfavorable comparisons.

By examining how parental behaviors influence adolescents’ tendencies to engage in upward social comparisons and how these behaviors, in turn, affect self-esteem, this research seeks to elucidate the pathways through which family dynamics can impact adolescent psychological well-being. Additionally, this study aims to explore the potential of optimism as a protective factor that could buffer the adverse effects of social comparisons. Through this exploration, the research intends to contribute to the development of targeted strategies that can enhance optimism among adolescents, thereby improving their self-esteem and overall mental health in the context of parental and peer comparisons.

To address these gaps, this study aims to investigate the relationship between parents’ social comparison and adolescents’ self-esteem, focusing on the mediating role of upward social comparison and the moderating influence of optimism. The following hypotheses are proposed:

*H1*: Parents’ social comparison is negatively associated with adolescents’ self-esteem.

*H2*: Upward social comparison mediates the relationship between parents’ social comparison and adolescents’ self-esteem.

*H3*: Optimism moderates the relationship between upward social comparison and self-esteem, such that the negative association is weaker for adolescents with higher levels of optimism.

## 2 Methods

### 2.1 Samples

Participants were selected using a convenience sampling method from a junior high school in Guangzhou, Guangdong. The school was chosen based on accessibility and willingness to participate in the study. All junior high school classes were approached, and students were invited to participate voluntarily. To ensure a representative sample, efforts were made to include students across different grades and academic performance levels. Informed consent was obtained from both students and their legal guardians prior to data collection. A total of 579 students were initially approached, employing a convenience sampling method due to the logistical ease of accessing participants within a single educational institution. Convenience sampling was chosen for this study as it allows for the efficient collection of data from a population that is readily accessible, thereby facilitating the examination of the research questions within a specific, confined context. After a preliminary screening of the survey responses, 3 samples were excluded due to uniform responses across multiple items, which could indicate a lack of attention or misunderstanding of the survey instructions. The final sample thus included 576 students. The gender distribution was relatively balanced with 294 males (51%) and 282 females (49%). The participants varied in age, with an average age of 14.32 years. The distribution across grades was 24 first-year students (4.2%), 147 s-year students (25.5%), and 405 third-year students (70.3%). This study utilized an online questionnaire platform where all items were mandatory, ensuring that no responses were missing. Additionally, the platform was designed to protect participant privacy.

### 2.2 Measurement

#### 2.2.1 Parents’ social comparison

The parents’ social comparison was measured using the Parental Social Comparison Scale revised by Chen (2022). This scale has a total of 7 questions, which are scored on a 5-point scale from 1 (strongly disagree) to 5 (strongly agree), with questions 5 and 7 scored in reverse order. The higher the total score, the higher the degree of social comparison perceived by adolescents with their parents. The fit index of confirmatory factor analysis was good: *χ*2/df = 6.56, RMSEA = 0.098, CFI = 0.97, NFI = 0.93, GFI = 0.92, TLI = 0.95, indicating that the revised scale has good structural validity. In this study, Cronbach’s *α* of this scale was 0.815.

#### 2.2.2 Upward social comparison

The upward social comparison subscale of the Iowa-Netherlands Comparison Orientation Measure compiled by Gibbons and Buunk (1999) was used as translated by Gibbons and Buunk (1999) and Xuejun et al. (2013). In order to make the measurement content more targeted, the comparison scope in the scale is limited to the situation of “comparing with other children.” This scale has 6 items (for example, “When I am told that other people’s children are better than mine, I compare myself with those ‘other children’.”). It is a 5-point Likert scale. The higher the score, the higher the score. The more frequently individuals make upward social comparisons with other children. Confirmatory factor analysis showed good structural validity: χ2/df = 18.56, RMSEA = 0.175, CFI = 0.92, NFI = 0.95, GFI = 0.97, TLI = 0.87, indicating that the revised scale has good structural validity. In this study, the alpha coefficient of this scale was 0.892.

#### 2.2.3 Self-esteem

The Chinese version of the Rosenberg Self-Esteem Scale was used for this study (Rosenberg, 1965; Xiangdong et al., 1999). The Chinese version of the Rosenberg Self-Esteem Scale originally included 10 items (e.g., “I feel that I am a valuable person, at least as good as others”), using a 4-point scoring system, with 1 indicating “very inconsistent,” 4 means “very consistent,” and the higher the score, the higher the individual’s self-esteem level. However, due to the cultural differences in the connotation of question 8 “I hope I can win more respect for myself,” many studies have deleted it when using this scale to improve the reliability and validity of the scale (Chen et al., 2013; Lumei, 2006; Shen and Cai, 2008). Research has also adopted this approach. The structural fitting coefficients of the questionnaire are χ2/df = 17.96, RMSEA = 0.172, CFI = 0.873, NFI = 0.908, GFI = 0.885, TLI = 0.83. In this study, the *α* coefficient of the scale is 0.925.

#### 2.2.4 Optimism

The life orientation questionnaire (LOT-R, LifeOrientation Test) compiled by Zhijun and Huichang (2007) revised by Scheier et al. (1994). The questionnaire contains a total of 12 items, including 5 items in the optimistic dimension (e.g., “I always feel that my luck will be very good”), and 5 items in the pessimistic dimension (e.g., “No matter how hard I try, I do not think things will go smoothly”), and the other two interference items are not included in the total score. A 5-point scoring method is used, with 1 representing “strongly disagree” and 5 representing “strongly agree.” After reverse-scoring the pessimistic items and adding the optimistic item scores, the individual’s overall optimism level is obtained. The higher the score, the higher the individual’s overall optimism level, the higher the level of optimism. The structural fitting coefficients of the questionnaire are X2/df = 10.37, RMSEA = 0.128, CFI = 0.859, NFI = 0.878, GFI = 0.862, TLI = 0.811, The alpha coefficient of this questionnaire is 0.855.

### 2.3 Process and data analysis

The administration of the study was conducted by main examiners, who were all teachers at the school from which the research sample was drawn. Utilizing classes as the basic units for data collection, each class was assigned a designated examiner to ensure standardized administration and oversight. The research utilized an online questionnaire platform; examiners distributed a link to the questionnaire to the participants in their respective classes. At the beginning of the questionnaire, a set of unified guidelines was provided. These guidelines clearly outlined the purpose of the study, detailed instructions on how to respond to the questionnaire, and emphasized the rights of the participants, including confidentiality and the voluntary nature of their participation. Participants were instructed to complete the questionnaire truthfully and independently to ensure the authenticity of their responses. To maintain the integrity of the data, questionnaires that were completed in a routine or cursory manner were identified and subsequently excluded from the final dataset. This step was crucial to ensure that the responses analyzed were thoughtful and representative of genuine participant perspectives.

Data analysis was conducted using SPSS 22.0. Descriptive statistics, correlation analysis, and common method bias testing were performed. A one-way ANOVA and independent sample t-test were conducted to examine group differences across demographic variables. This study employed a moderate mediation model to examine the interplay between parents’ social comparison, upward social comparison, optimism, and adolescent self-esteem. The proposed model hypothesizes that upward social comparison mediates the negative relationship between parents’ social comparison and self-esteem, while optimism moderates the indirect path between upward social comparison and self-esteem. The model aligns with the PROCESS macro (Model 59) by Hayes (2012).

### 2.4 Ethical considerations

Prior to the commencement of this study, ethical approval was secured from the Institutional Review Board (IRB) of the relevant institution, ensuring all research protocols adhered to the ethical standards required for studies involving human subjects. Informed consent was obtained from all participants and their legal guardians. This consent process involved providing detailed information about the study’s purpose, the nature of participation, the voluntary basis of involvement, and the right to withdraw at any time without penalty. Participants and guardians were assured of their right to confidentiality and were informed about how the data would be used and protected. To protect participant privacy, all data collected were anonymized. Identifiable information was removed or encrypted, and only the research team had access to the raw data. Results are reported in aggregate form, with no possibility of tracing back to individual participants. The study was designed to minimize psychological distress. The questionnaires used were non-invasive and posed no risk of psychological harm. Participants were informed that they could skip any questions that they felt uncomfortable answering and could stop participating in the study at any time. Given the cultural context of the participants, the study was designed and conducted in a manner that respects and acknowledges cultural norms and values, particularly regarding family dynamics and educational pressures in the Chinese context.

## 3 Result

### 3.1 Common method bias test

To ensure the validity of our findings, it was necessary to address the possibility of common method bias, which can occur in studies utilizing self-report questionnaires (Podsakoff et al., 2003). We employed Harman’s single-factor test, a widely recognized method for detecting this type of bias (Harman, 1976). This procedure involved conducting an exploratory factor analysis on all items in the questionnaire to determine the amount of variance that could be attributed to a single factor. The analysis revealed that the first factor accounted for only 34.14% of the total variance, which is significantly below the 40% threshold commonly used to suggest a meaningful level of common method bias (Podsakoff et al., 2003). Based on these results, common method variance does not appear to substantially contaminate the data, lending credence to the integrity of the conclusions drawn from this study.

### 3.2 Difference statistics and correlation analysis

The results of the independent sample *t-*test show that there are significant gender differences in optimism (*t* = 3.592, *p* < 0.01, *d* = 0.29), with boys having slightly higher optimism than girls. One-way ANOVA found that there were grade differences in upward social comparison tendency [*F*(2,573) = 4.58, *p* < 0.05, η2 = 0.016]. The upward social tendency of first-year junior high school students (*M* = 13.37, SD = 4.24) was significantly lower than the second grade (*M* = 15.17, SD = 6.07) and the third grade of junior high school students (*M* = 16.25, SD = 5.38). Upward social comparison tendencies tend to increase as grades increase. In addition, there are grade differences in optimism [*F*(2,573) = 5.42, *p* < 0.01, η2 = 0.019]. The optimism of first-year junior high school students (*M* = 36.12, SD = 7.12) is significantly higher than second-year junior high school students (*M* = 32.81, SD = 6.30) and third grade of junior high school students (*M* = 31.89, SD = 6.52), optimism has a downward trend as grades increase. For the sake of conservatism, gender and grade were used as control variables in subsequent analyses to eliminate their effects. The correlation analysis results after controlling for gender and grade show: Parental social comparison showed a significant positive correlation with upward social comparison and a significant negative correlation with self-esteem and optimism. Upward social comparison was significantly and negatively related to self-esteem and optimism. There is a significant positive correlation between self-esteem and optimism (see Table 1).

**Table 1:** Correlation analysis results between variables.

Variables	*M*	SD	1	2	3	4
1. Parents' social comparison	20.922	5.781	1			
2. Upward social comparison	15.851	5.563	0.472***	1		
3. Self-esteem	25.621	5.621	−0.396***	−0.340***	1	
4. Optimism	32.303	6.544	−0.427***	−0.279***	0.651***	1

*p* < 0.05, ** *p* < 0.01, *** *p* < 0.001, all values are rounded to three decimal places, the same below.

### 3.3 Moderated mediating effect test

Using the Model 59 of SPSS macro program PROCESS of Hayes (2012), and controlling for gender and grade, to analyze whether the mediating role of upward social comparison between parents’ social comparison and self-esteem is moderated by optimism. Testing whether the mediating role of upward social comparison between parents’ social comparison and self-esteem is moderated by optimism, the results show (as shown in Table 2): Parents’ social comparison significantly and positively predicts upward social comparison (*β* = 0.469, *p* < 0.001); Upward social comparison significantly and negatively predicts self-esteem (*β* = −0.151, *p* < 0.001); The predictive effect of parents’ social comparison on self-esteem is only less significant (*β* = −0.081, *p* < 0.05); The interaction term of upward social comparison and optimism has a significant positive predictive effect on self-esteem (*β* = −0.045, *p* < 0.05).

**Table 2:** Hayes’ Model analysis results of optimism mediating the mediating role of upward social comparison between parents’ social comparison and self-esteem.

Equation		Overall fit index			Model coefficient significance			
Outcome variable	Predictor variable	*R*	*R*2	*F*	β	95% CI lower	95% CI upper	*t*
Upward social comparison	Gender	0.489	0.238	59.66***	0.069	−0.003	0.1404	1.873
	Grade				0.088	0.015	0.159	2.379
	Parents’ social comparison				0.469	0.397	0.541	12.802***
	PSC × OP				0.007	−0.052	0.066	0.234
Self-esteem	Gender	0.678	0.459	80.456***	0.037	−0.025	0.098	1.175
	Grade				0.036	−0.026	0.097	1.137
	Parents’ social comparison				−0.081	−0.155	−0.007	−2.147*
	Upward social comparison				−0.151	−0.222	−0.08	−4.175***
	Optimism				0.58	0.511	0.649	16.648***
	PSC × OP				0.029	−0.032	0.089	0.928
	USC × OP				−0.045	−0.102	0.012	−1.5443*

PS: PSC, parents’ social comparison; OP, optimism; USC, upward social comparison. Each variable in the model is standardized and then brought into the equation, the same as below.

Structural equation modeling (SEM) was employed to test the hypothesized moderated mediation model. This approach allowed for a more comprehensive assessment of the direct, indirect, and moderating effects. Additionally, bootstrapping analysis (5,000 samples) was conducted to estimate the indirect effects and generate confidence intervals. The bootstrapped results confirmed the significance of the mediation and moderation pathways, further supporting the robustness of the proposed model. When the optimism score is the mean minus one standard deviation, the mean, and the mean plus one standard deviation, the mediating effect value of upward social comparison between parents’ social comparison and self-esteem and its 95% Bootstrap confidence interval are as follows as shown in Table 3.

**Table 3:** The mediating effect of upward social comparison between parents’ social comparison and self-esteem at different levels of optimism.

Optimism	Mediating effect size	SE	95% CI Lower	95% CI upper
M – SD	−0.108***	0.048	−0.202	−0.114
*M*	−0.079**	0.038	−0.153	−0.065
M + SD	−0.050*	0.051	−0.148	−0.006

Based on the above results, the moderated mediation model proposed in this study is supported. Parents’ social comparison has a direct predictive effect on self-esteem, and upward social comparison plays a mediating role between parents’ social comparison and self-esteem, and the second half of this mediating effect (upward social comparison → self-esteem) is moderated by optimism.

**Figure 1:**
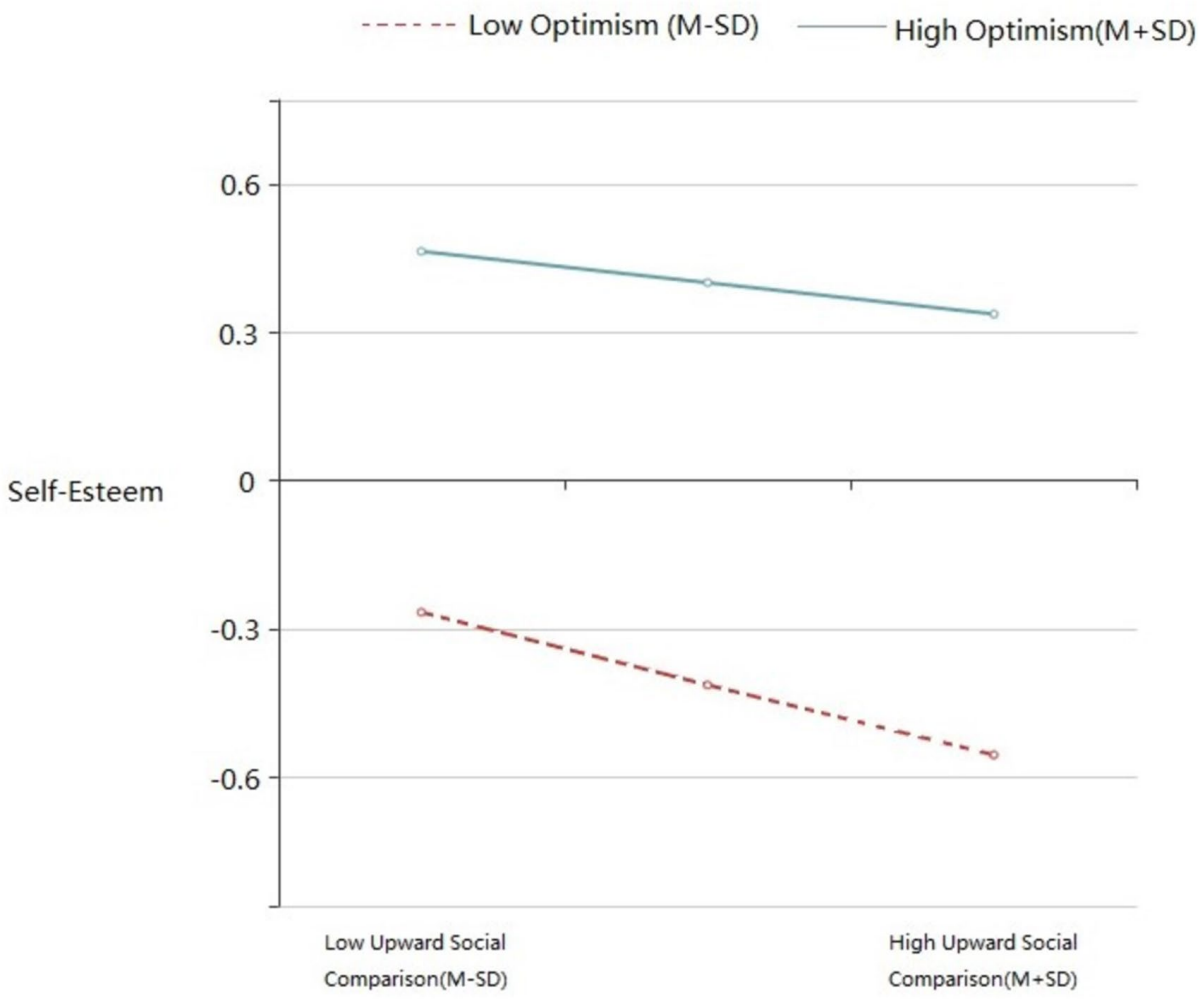
The moderating effect of optimism on the relationship between upward social comparison and self-esteem.

A simple slope test was further used to analyze the moderating role of optimism in upward social comparison and self-esteem. The subjects were divided into high optimism level group (subjects who were above the mean plus one standard deviation) and low optimism group (subjects who were below the mean minus one standard deviation) according to the average optimism score plus or minus one standard deviation and the moderate optimism group (subjects between the above two groups). A simple slope test was used to examine the relationship between upward social comparison and self-esteem. The results are shown in Figure 1: As the level of optimism increases, the negative predictive effect of upward social comparison on self-esteem gradually weakens, but its effect is still significant (from *β* = −0.336, *p* < 0.001 to β = −0.072, *p* < 0.05).

## 4 Discussion

The findings reveal that parents’ social comparison negatively impacts adolescent self-esteem both directly and indirectly, via the mediation of upward social comparison. Consistent with social learning theory (Bandura and Walters, 1977), the results suggest that adolescents may internalize the social comparison behaviors exhibited by their parents. When parents frequently engage in comparisons that implicitly or explicitly deem their children less competent or less successful than others, it may directly diminish adolescents’ views of their self-worth, thus directly lowering their self-esteem. This finding aligns with previous research indicating that parental behaviors and attitudes significantly shape children’s self-perceptions and psychological outcomes (Candel, 2022; Gentzler et al., 2010; Pettit et al., 2001).

### 4.1 The mediating role of upward social comparison

Previous research has found that information about “other people’s children” can significantly and positively predict upward social comparison (Liu, 2018). This study further shows that parents’ social comparison can enhance adolescents’ upward social comparison tendency with their peers, which is a further in-depth and detailed study of previous studies. Influenced by parenting behaviors in which parents constantly compare other people’s children to themselves, adolescents unconsciously reinforce the tendency to compare themselves to their peers. Past studies have shown that social comparison tendencies tend to arise in similar groups, such as similar age, living and learning environments (Buunk and Gibbons, 2006; Fassl et al., 2020). Thus, parenting behaviors such as parents’ social comparison trigger and reinforce this upward social comparison tendency in adolescents, and when parents continually emphasize that adolescents should compare themselves with those who are better than them rather than those who are less than them, adolescents only reinforce the upward social comparison tendency rather than the downward social comparison tendency. In short, parents’ social comparison affects both the tendency to make social comparisons (reinforcing the tendency to make comparisons) and the direction of social comparisons (inducing upward comparisons), so it is said that adolescents’ passive reception of information about “other people’s children” is an important reason for the induction of upward social comparisons by parents’ social comparisons.

Upward social comparisons can cause individuals’ self-evaluations to deviate significantly from the comparison goals and to be at levels significantly lower than the comparison goals, and individuals’ self-evaluations can become more negative as a result (Lee, 2022; Mann and Blumberg, 2022; Meier and Johnson, 2022). Consistent with social comparison theory and previous research, the present study also found that upward social comparison significantly and negatively predicted self-esteem (Bergagna and Tartaglia, 2018; Fagundes et al., 2020; Jiang and Ngien, 2020; Suls and Wills, 2024). Upward social comparison plays a partial mediating role between parents’ social comparison and adolescents’ self-esteem, implying that parents’ social comparison can influence adolescents’ self-esteem in different ways than just through the path of reinforcing adolescents’ social comparison tendencies. Firstly, it highlights the significant role parents play in shaping the social comparison orientation of their children. Secondly, it underscores the potential negative consequences of such comparisons. Adolescents, who are in a critical developmental phase of building self-identity and self-esteem, may be particularly vulnerable to the detrimental effects of these comparisons.

### 4.2 The moderating effect of optimism

**Figure 2:**
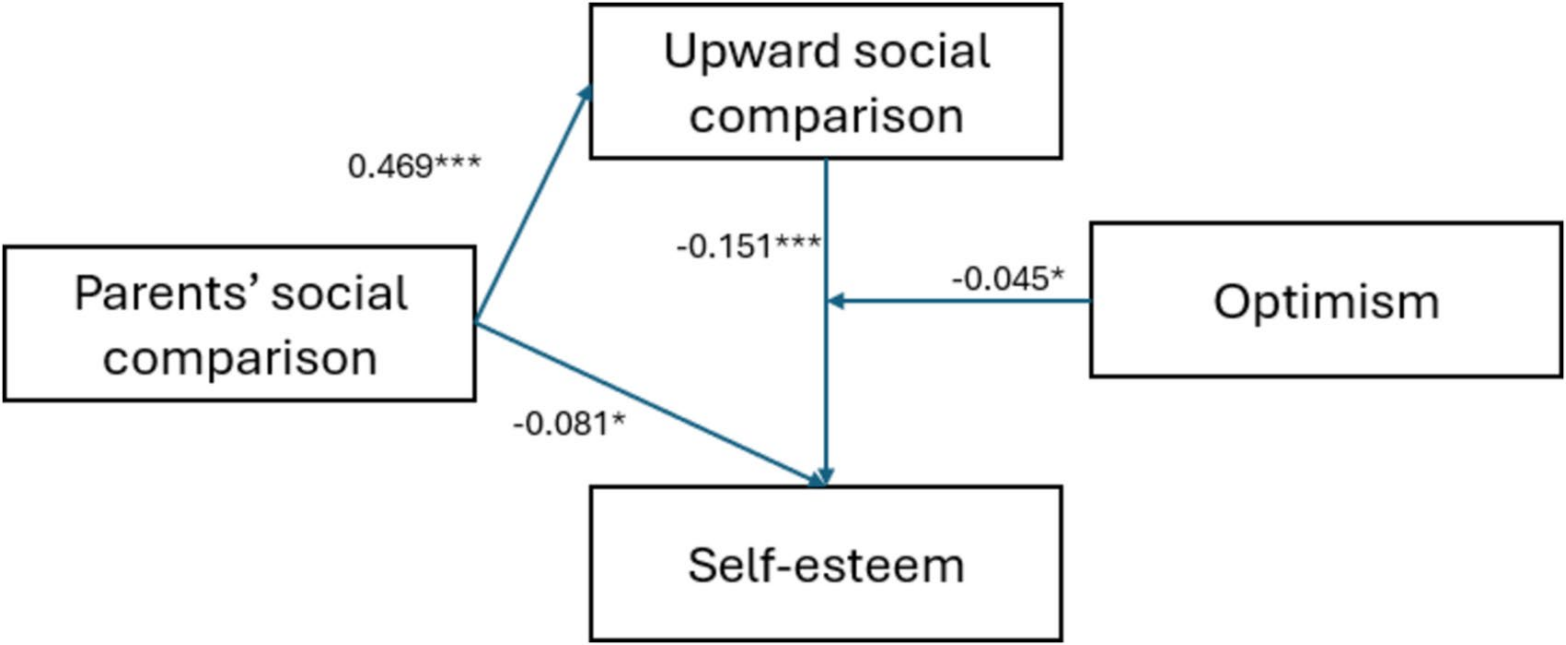
The moderated mediation model.

The present study also found that the mediating effect of upward social comparison between parents’ social comparison and self-esteem was moderated by optimism, and that the indirect effect was more significant in individuals with low levels of optimism relative to those with high levels of optimism. A moderate mediation model was thus formed (see Figure 2). Past research has demonstrated the protective effects of optimism on an individual’s mental health (Gallagher et al., 2020; Schug et al., 2021). That is, optimism as an important psychological resource can significantly mitigate the negative effects of negative factors on physical and mental health, and furthermore, it has been shown that optimism affects individuals’ processing and understanding of upward social comparison information, which in turn plays a good protective role for individuals (Gibbons and Murray-Gibbons, 2022).

From previous research on optimism in positive psychology, it can be found that optimism may affect individuals’ processing and understanding of social comparison information in three ways: cognitive, emotional, and motivational. Research by Scheier and Carver (1985), who developed the concept of dispositional optimism, suggests that optimists are more likely to expect positive outcomes and, therefore, may cognitively downplay negative comparisons or view them as opportunities for growth and learning (Scheier and Carver, 1985). As for the emotional aspect, research by Carver, Scheier, and Segerstrom found that optimists experience less distress and more positive emotional states in response to potentially stressful situations, including challenging social comparisons (Segerstrom et al., 2017). On a motivational level, optimism can drive individuals to respond to social comparison information by setting higher goals and sustaining effort toward achieving those goals. Optimists are more likely to use social comparisons as motivation to improve themselves rather than as a demoralizing factor. Aspinwall and Taylor discuss how optimists use adaptive coping strategies more frequently, such as goal-directed behavior and strategic planning, in response to challenges (Aspinwall and Taylor, 1997).

First, the attributional style theory of optimism suggests that optimism is an explanatory style in which high optimists tend to make enduring, general, and personal attributions for good things and transient, specific, and extrinsic attributions for bad things (Liu and Bates, 2014; Snyder and Lopez, 2001; Zhang et al., 2014). Thus, “others are better than oneself” may be seen by the high optimist as temporary, or better than oneself in only one aspect (Endo, 2007; Liu et al., 2017). According to the Extended-Construct Theory of Positive Emotions, positive emotions construct and enhance personal resources (Fredrickson, 2001; Xu and Chen, 2023; Yang et al., 2024), and relevant studies have confirmed that people with high levels of optimism have more psychological resources, such as hope, self-efficacy, and psychological resilience (D'Souza et al., 2023; D’Souza et al., 2020; Feldman and Kubota, 2015; Lamont et al., 2019). Resource Conservation Theory, on the other hand, suggests that individuals with more resources are better able to cope with stress and are happier and more joyful (Doane et al., 2012; Hobfoll et al., 1990; Merino et al., 2019), and that happier and more joyful people are better able to strategically comprehend socially comparative information (Alfasi, 2019; Kim et al., 2016; Lyubomirsky and Ross, 1997; Lyubomirsky et al., 2001). Third, individuals with high levels of optimism use proximity (problem solving) rather than avoidance coping strategies (Brandstätter et al., 2019; Rittichainuwat et al., 2018), and in the face of the pressure of upward social comparisons, individuals with high levels of optimism may try to shorten the gap between themselves and others, and the upward social comparisons induced by the parents’ social comparison may play a certain role in motivating individuals with high levels of optimism. Therefore, individuals with high optimism can respond to upward social comparison more positively, which may weaken or even inhibit the contrast effect of upward social comparison.

In addition, a meta-analysis of the relationship between optimism and psychological well-being indicated that among different positive indicators of psychological well-being (e.g., self-esteem, self-concept clarity, resilience, and subjective well-being, self-efficacy), optimism had the highest correlation with self-esteem (Qi et al., 2012), and thus, optimism can play a protective role for self-esteem.

### 4.3 Significance, limitations of the research and prospective

This research delineates a clear pathway through which parents’ social comparison behaviors can impact adolescent self-esteem. Specifically, the study reveals that when parents frequently compare their children to others, it often leads to increased upward social comparison among adolescents—a process where they measure themselves against peers who are perceived to be more successful or capable. This type of comparison can diminish self-esteem, especially if adolescents perceive these comparisons negatively. The addition of optimism as a moderating factor in this model suggests that adolescents with a positive outlook are more resilient to the potential negative effects of such comparisons. They are likely to perceive comparison information more constructively, potentially mitigating negative impacts on self-esteem (Carver and Scheier, 2017).

Theoretically, this study contributes to social comparison theory by integrating the role of familial influence and individual traits like optimism. It suggests that parental behaviors not only initiate comparison tendencies but also shape the way these comparisons are internalized and processed by adolescents. This underscores the dynamic nature of social comparison, influenced by both environmental cues and personal dispositions.

Practically, in response to the findings of the study on the impact of parental social comparisons on adolescent self-esteem, several practical implications and educational recommendations have been proposed to address the identified challenges. Firstly, the development and implementation of parenting workshops are crucial. These workshops should educate parents about the negative psychological impacts of unfavorable social comparisons and encourage practices that focus on recognizing and reinforcing the unique strengths and abilities of their children. This approach promotes a supportive family environment that celebrates individual achievements rather than fostering competitive comparisons. Secondly, enhancing optimism in adolescents through school and community programs can serve as a buffer against the negative effects of social comparison. Programs that include training sessions on positive thinking, resilience building, and activities that promote self-efficacy and personal growth are recommended. Such initiatives help adolescents to view social comparisons more constructively and maintain healthy self-esteem. Additionally, schools should ensure that counseling services are readily available to assist students who struggle with issues related to self-esteem and social comparison. These services should offer both individual counseling to address personal feelings of inadequacy and group therapy sessions that provide a platform for students to share experiences and learn coping strategies. Moreover, integrating lessons on emotional intelligence, social skills, and self-awareness into the educational curriculum is essential. This integration helps students to manage their emotions effectively and maintain a healthy self-image in a competitive social environment. Educators should also be vigilant and proactive in identifying signs of distress among students, which may indicate issues with self-esteem linked to negative social comparisons.

Despite its contributions, this study has several limitations that need acknowledgment. First, the use of cross-sectional data limits our ability to infer causality between parents’ social comparison, upward social comparison, optimism, and self-esteem. Longitudinal studies are recommended to confirm the directionality of these relationships. Second, the study relies heavily on self-reported data, which might introduce bias such as social desirability or response bias. Future research could benefit from incorporating more objective measures or multi-source data to validate the findings. Additionally, while the sample provides a good representation of adolescents from a specific cultural and educational setting, the findings may not generalize to adolescents from different backgrounds or cultural contexts. Further studies involving diverse demographic groups are necessary to examine the applicability of these findings across various populations. The uneven grade distribution, with a higher concentration of third-year students, poses a potential limitation. Despite controlling grades in the analyses, future studies should aim for more evenly distributed samples to avoid overrepresentation of specific age groups. Finally, this study relied on self-report measures from adolescent participants, which may introduce biases inherent in single-informant designs. While this approach provides valuable insights into adolescents’ perceptions, future research should consider employing a multi-informant approach, including parent-student days or teacher evaluations, to enhance the robustness of the findings. This limitation may reduce the generalizability of results and warrants cautious interpretation.

Building on the foundations laid by the current study, several directions for future research have emerged, aiming to deepen the understanding of the dynamics between parental social comparison, upward social comparison, optimism, and adolescent self-esteem. First, future studies should consider employing longitudinal research designs. Such designs would allow researchers to track changes over time, providing insights into the causality and directionality of the relationships identified in this study. This approach could help determine whether parental social comparisons have long-term effects on adolescent self-esteem and whether these effects change as adolescents grow older.

Second, to enhance the reliability and depth of data, future research could incorporate a multi-method approach that goes beyond self-reported measures to include observations, diary methods, or interviews. These methods could reduce potential biases inherent in self-reports, such as social desirability bias, and provide a more nuanced understanding of the internal states and real-time dynamics of social comparison processes.

Additionally, expanding the research to include diverse cultural and demographic contexts would be invaluable. Given that cultural norms significantly influence parenting styles and perceptions of self-worth, studies in diverse settings could determine the universality or specificity of the findings. This could involve cross-cultural studies that compare how social comparison dynamics operate in different cultural contexts, which might reveal unique protective factors or vulnerabilities.

Lastly, integrating qualitative research could offer deeper insights into the personal experiences and narratives of adolescents dealing with social comparison. Qualitative studies can explore the subjective interpretations and meanings that adolescents attach to their experiences with parental comparisons and how these influence their self-esteem and coping mechanisms. Such insights could be instrumental in developing targeted interventions tailored to the nuanced needs of adolescents facing these challenges.

## Data Availability

The raw data supporting the conclusions of this article will be made available by the authors, without undue reservation.
